# Feasibility and acceptability of a new web-based cognitive training platform for cognitively healthy older adults: the breakfast task

**DOI:** 10.1186/s40814-023-01359-2

**Published:** 2023-08-04

**Authors:** Sharon Sanz Simon, Daniel Ben-Eliezer, Maria Pondikos, Yaakov Stern, Daniel Gopher

**Affiliations:** 1 Taub Institute for Research in Alzheimer’s Disease and the Aging Brain, Columbia University, New York, NY USA; 2 Cognitive Neuroscience Division, Department of Neurology, Columbia University, New York, NY USA; 3 Faculty of Industrial Engineering and Management Technion, Israel Institute of Technology, Technion city, Haifa 32000 Israel

**Keywords:** Clinical trial methods, Digital intervention, Healthy/active aging, Cognition, Multitasking

## Abstract

**Background:**

Developing efficient cognitive training for the older population is a major public health goal due to its potential cognitive benefits. A promising training target is executive control, critical for multitasking in everyday life. The aim of this pilot study was to establish the feasibility and acceptability of the Breakfast Task training in older adults, a new web-based cognitive training platform that simulates real-life multitasking demands.

**Methods:**

A community-based sample of 24 cognitively healthy participants aged between 60 and 75 (*M* = 69.12, SD = 3.83) underwent 5-session cognitive training protocol, delivered online. Each session lasted 45 min and occurred twice a week at participant’s homes. Performance was recorded, and participants completed questionnaires at baseline and after the intervention.

**Results:**

Feasibility metrics showed overall high recruitment (82.7%), adherence and retention rates (100%). Acceptability was considered good based on participant’s quantitative and qualitative responses. On average, participants rated the game as interesting, enjoyable and did not report difficulties in accessing the game online without supervision or in understanding the instructions. Participants showed a learning curve across sessions, suggesting improvement in the game outcomes and potential benefits from the emphasis change training approach. The study identified relevant areas that need improvements and adjustments, such as technical issues, session’s structure, and dose.

**Conclusions:**

The findings provide preliminary support for the feasibility and acceptability of the web-based Breakfast Task training platform in cognitively healthy older adults. Results suggest the value of further research to investigate the Breakfast Task training features and dose-response relationship, as well as its potential efficacy in older adults via larger randomized controlled trials.

**Trial registration:**

ClinicalTrials.gov: NCT04195230 (Registered 11 December 2019).

## Key messages regarding feasibility

- Acceptability is good but variable
- Technical issues and dose still need to be adjusted
- Good recruitment rate (82.7%), great adherence and retention (100%) in a short training regimen, and presence of learning curve across sessions
- The results support the use of the Breakfast Task platform and emphasis change training approach in future trials

## Background

Maintaining a cognitively active lifestyle has been associated with better cognitive function and reduced dementia risk in late-life [19, 22, 27, 36, 38, 44, 48]. Therefore, a major public health goal is developing effective cognitive interventions to enhance and/or maintain cognitive health in older adults, which may attenuate age-related cognitive decline and contribute to functional independency, quality of life, and dementia prevention.

A critical intervention approach for older adults is cognitive training, or the repeated practice of standardized exercises targeting specific cognitive processes that may optimize cognitive functioning in everyday life [22, 41]. In older adults, cognitive training has been associated with short-term cognitive improvements and near-transfer effects [1, 4, 6, 13, 14, 17], Lampit, Hallock, and Valenzuela, 2014, [36, 40, 42, 43, 50], as well as some long-term benefits, such as reduction of cognitive/functional decline [2, 38] and dementia risk [19], although more evidence is needed for definitive conclusion. It is hypothesized that cognitive training in older adults may induce brain processes that protect individuals from the effects of aging and brain diseases, possibly by increasing cognitive reserve later in life [45, 46].

Technology has been increasingly integrated to cognitive training and is an opportunity to improve intervention design, engagement, and accessibility to cognitive intervention. Different reviews indicate that computerized cognitive training is feasible in cognitively heathy older adults [6, 31, 40, 43], and there is evidence that the same cognitive training protocol can show similar effects when delivered remotely (online) or face-to-face [39]. One of the main challenges is the limited transfer effects, which typically occurs to proximal outcomes, but not to distal outcomes (i.e., far-transfer or context transfer) [6, 37, 40, 43]. The limited transfer effects suggest the need of training protocols that are more meaningful and related to everyday life demands, which can facilitate transfer to distal outcomes. In addition, there is evidence that unsupervised online cognitive training is less effective than supervised intervention [31], a critical ingredient to consider when delivering online interventions. Another challenge when delivering computerized cognitive training online is adherence, not always easy to predict, but it seems to be facilitated through intervention reminder system [26] and a promising approach is hybrid supervision design, where sessions are partially supervised remotely.

A relevant target for cognitive training is executive control, which is known to decline with aging and is critical for multi-tasking in everyday life [1, 9, 10, 49] (e.g., talking while driving, cooking a meal for guests). Despite the challenge demonstrating training-transfer effects in older adults, executive control training has shown encouraging findings. For instance, executive control training programs [1, 7, 8, 10, 12, 23, 24, 34, 47, 51] have shown improvements in performance on the trained tasks and “near”/content transfer effects, reflecting transfer of gains from trained tasks to untrained tasks of similar nature or content [5]. However, the extent to which transfer occurs to a different or more distal context (far or context-transfer), such as everyday life situations, remains unclear [6, 37, 40, 43].

A promising executive control/multitasking training approach is emphasis change (EmCh), a method that requires participants to systematically change their emphasis/attention allocation policy between subcomponents of a task, enhancing exploration of solution strategies and cognitive flexibility [23]. EmCh has demonstrated context-transfer from a complex video game to actual flight performance in young pilots [24]. The same protocol enhanced executive functions in older adults,however, the motor requirements of the task limited the results in a subset of participants [12, 47]. A similar training method, Variable Priority, has endorsed the EmCh findings by showing that multi-tasking performance is optimized when attention is prioritized towards one task over the other, i.e., when emphasis change instructions are utilized [11, 30, 33]. These training methods are considered particularly powerful for inducing transfer of training in older adults [10]. Variable priority studies have found age-equivalent content-transfer effects among young and older adults [8, 33], and even larger transfer effects in older adults [11], in contrast to other training approaches with limited transfer in older population [17, 18].

To date, the EmCh approach has not been applied to ecological tasks, which simulate daily life situations, and may enhance its clinical and real-life relevance. Moreover, EmCh has not been implemented remotely, which may facilitate its accessibility to a diverse population (e.g., individuals with mobility difficulties, living in remote regions, and across education backgrounds, genders, and race/ethnicities) and adaptability to different life demands. Strategies to keep older adults cognitively active at home are additionally relevant after the COVID-19 pandemic, which may limit in-person social interaction and research participation.

The aim of this pilot study is to establish the feasibility and acceptability of the EmCh approach using a new ecological web-based cognitive training platform, the Breakfast Task (BT), among cognitively healthy older adults. We hypothesized that older adults would be able to engage and use the training platform without supervision, consider it acceptable, and show some responsiveness to the EmCh approach.

## Methods

### Study design

**Fig. 1:**
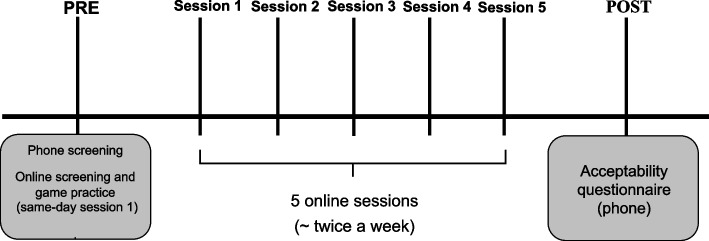
Study design

The overall study design is illustrated in Fig. 1 and includes a brief pre-intervention screening, five sessions, and post-intervention questionnaire. This single-arm online interventional study occurred during the COVID-19 pandemic, from January to July 2021. It is worth mentioning that this study was originally conceptualized to occur in-person and was initiated at the end of 2019. However, due to the pandemic, the study was interrupted in March 2020, the methods adjusted to be remote/online, and the study was re-initiated in 2021. The limitation of in-person interactions imposed by the pandemic was an opportunity to integrate telehealth to the project and collect data online as participants participated in the study from their homes. The CONSORT 2010 guideline for pilot and feasibility trials [20] was used to report the findings of this study. Items that were not applicable such as randomization and blinding were omitted as there was no control group.

### Registration, ethics approval, and online consent

The trial was registered on the *ClinicalTrials*.gov registry (ID: NCT04195230). The study protocol and documents were reviewed and approved by the Internal Review Board of the College of Physicians and Surgeons of Columbia University (reference: IRB-AAAS6529). Online written informed consent to participate was obtained from each participant through a secure electronic signature system.

### Recruitment and eligibility

Participants living in the New York City area were recruited from the community via flyers, emails to NGOs focused on aging populations, referrals from colleagues and research subjects, and Columbia University’s online recruitment platform (*RecruitMe*), which connects university researchers to potential participants. It is worth mentioning that initially this was an in-person study, and part of the sample were contacted before the COVID-19 pandemic (October–December 2019). However, due to the pandemic, we adapted the study to be online and in February 2021 we re-initiated the study e re-contacted the participants.

Although formal sample size calculation may not be appropriate for pilot studies [29], due to the online and remote nature of this intervention, and potential loss of participants, we recruited more participants than the usual rule of thumb for pilot studies (12 per group) [29]. Our group size was larger than our previous in-person EmCh study [47] and the minimum recommended (*n* = 20) by a comprehensive review on computerized brain-games [43].

At pre-training, participants underwent a telephone screening to collect data about demographics and medical history along with a brief remote cognitive and functional screening (on Zoom). To be included in the study, participants had to meet the following eligibility criteria: age between 60 and 75 years; have the capacity to speak and read in English; preserved or corrected vision and hearing; preserved cognitive performance on the remote Montreal Cognitive Assessment (MoCA) (score ≥ 26) [15, 28, 35] and on the Activities of Daily Living-Extended (ADL-x) scale (score > 5) [21]. In addition, participants had to be able to navigate on internet, use a computer (desktop or laptop) and a mouse. Smartphones and tablets were not allowed, due to screen size. In the case participants had difficulty using the Zoom or did not have access to a computer/internet, the study team provided Zoom tutorial/practice and equipment with internet. Participants were excluded if they present any major neurological or psychiatric conditions or use of medication considered to affect cognition.

### Intervention

#### Breakfast task training platform: development and translation

**Fig. 2:**
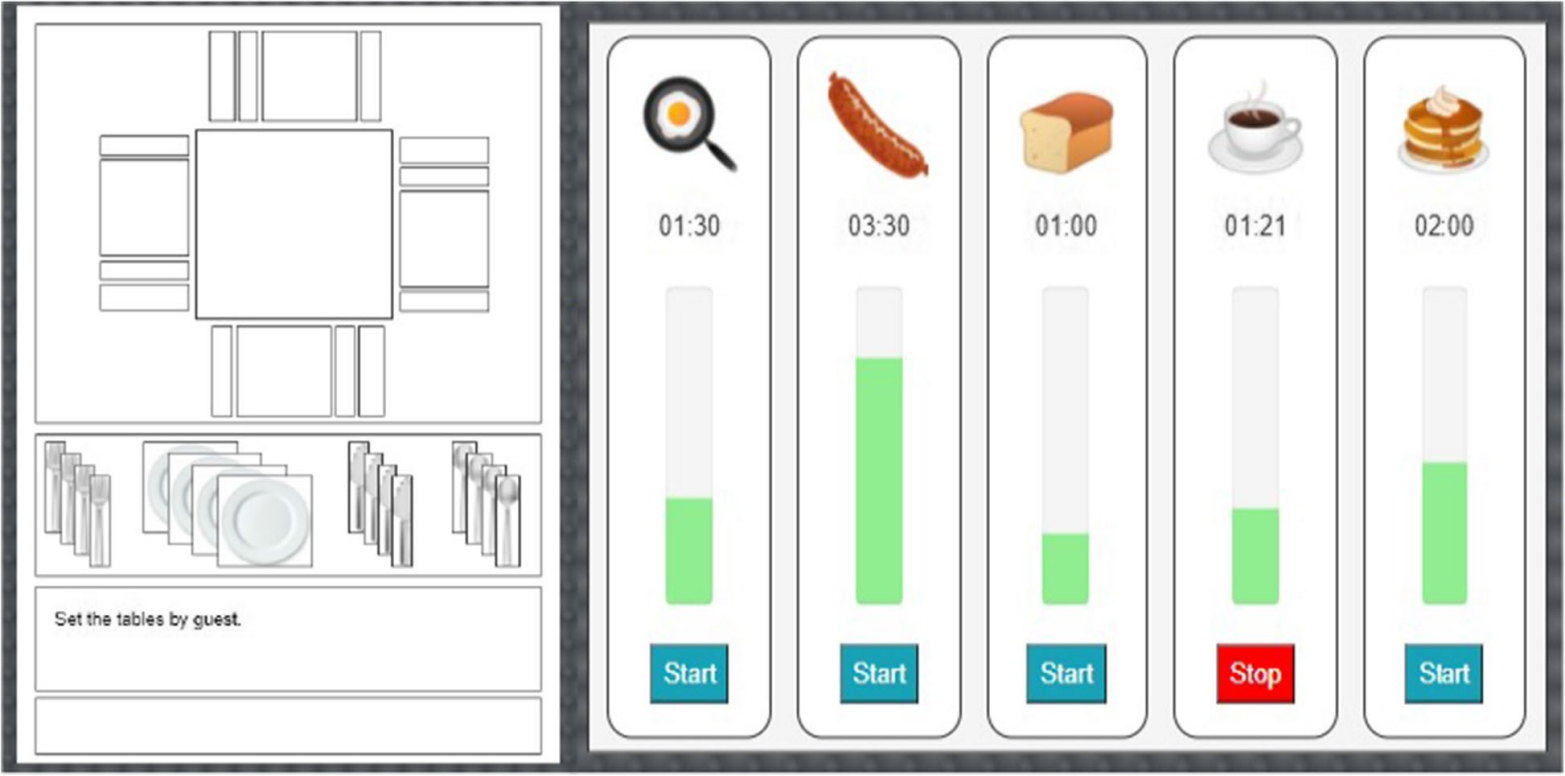
Breakfast Task Training illustration. *Legend:* Breakfast Task training platform showing both tasks in the same screen. On the left side, the Table Setting task: the table and four guests seats are displayed with space for plates and utensils. Each participant must set the tables by guest or tableware rule. On the right, the Cooking task: food items are displayed with the cooking time in minutes and seconds and illustrated with bars. Participants must press “start” to begin cooking and “stop” to finish cooking

The BT training is a new computerized game platform designed by Gopher et al. [25] based on the Breakfast Task, a well-established computer-based task developed to evaluate executive control in older adults [16]. In 2020, during the COVID-19 pandemic, the BT was adapted to a web-based format, enabling access from participant’s homes. In brief, the BT simulates a life situation that demands executive control, attention management, and multi-tasking. It includes two simultaneous tasks: the Table Setting task, in which participants have to set tables for guests, and the Cooking task, in which participants have to cook foods with different cooking time requirements (Fig. 2). Some advantages of the BT platform are (1) the training can be designed under different instructions as well as difficulty levels, (2) flexibility in terms of duration and number of trials or sessions, (3) automated scoring system, and (4) the platform is accessed via the internet, hence can be administered remotely.

The BT instructions were first created in Hebrew at the Technion Institute of Technology, then translated to American English in collaboration with Columbia University, in three steps. First, two English-Hebrew speaking people produced an independent translation from Hebrew to English. Second, these versions were unified to reach a consensus on the English version under the supervision of two neuropsychologists trained in the aging field. Third, to ensure the accuracy of the translation, the training instructions were translated back from English to Hebrew by a neuroscience student proficient in both languages.

#### Game tasks: table setting and cooking

During the game, participants were instructed to “prepare breakfast”, so they set tables for guests while concurrently cooking foods for breakfast. The game goals were to: (1) set as many tables as possible, (2) cook each food item in its accurate time (i.e., not over- or under-cooking), and (3) finish cooking all food items at the same time so they could be served together. Scoring measures combined performance on the Table Setting and Cooking tasks, and the overall goal was to achieve a better point score for both tasks.

In the Table Setting segment, participants were asked to set a table for four guests by placing plates, forks, knives, and spoons in the appropriate location on each placemat. Simulating the Western etiquette frequently used, participants were instructed that forks should be set on the left side of the plate, knives to the right side of the plate, and spoons on the right side of the knives. The program did not allow placement of a tableware in an incorrect location and only allowed placement in the aforementioned places. Participants were instructed on these rules and did a practice trial prior to starting the scored session. In each given round, participants were instructed to set tables according to one of two table-setting rules: (1) *by guest*, meaning the complete tableware set should be placed for one guest before moving onto the next guest, or (2) *by tableware*, meaning each type of tableware should be placed at once for all four guests (i.e., set all four forks, then set all four knives). Table setting scores counted only fully set tables following the correct rule.

In the Cooking segment, participants were asked to cook two to five food items. To start cooking, participants had to press “start” under each food item displayed and press “stop” when they finished cooking each food. Cooking times were displayed in minutes for each item and are always the same throughout the training (coffee: 4.5, sausage: 3.5, pancakes: 2, egg: 1.5, and toast: 1 min). Participants started each trial by starting the food item with the longest cooking time (i.e., the coffee). Ideally, each food item should be started and stopped at the accurate times so they all finished at the same time and could be served together. For the foods to be accurately cooked and for all items to be completed together, each subsequent food should be started when the time remaining for the foods being cooked was equivalent to the food’s cooking time, meaning that the cooking timer should be started for the longest food cooking time first and the shortest food cooking time last.

In some sessions, both the Table Setting and Cooking tasks were equally important toward scoring, and participants were therefore expected to divide their attention equally between tasks. In other sessions, however, EmCh was applied, and participants were asked to prioritize one task over the other while playing the game. Under EmCh, participants received the instruction to pay special attention to one of the tasks and were told that 75% of their scores would be based on the performance of the emphasized task.

#### Intervention design

**Fig. 3:**
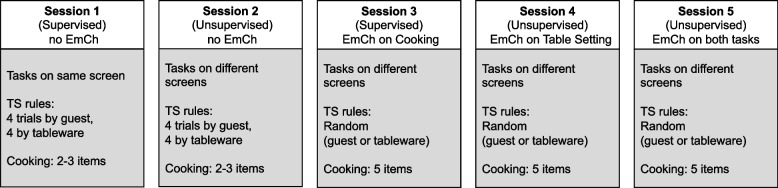
Training design and features. *Legend*. EmCh: Emphasis Change; TS: Table Setting

The BT training design consisted of total five individual sessions delivered twice a week. Each session lasted approximately 45 min and was comprised of eight trials lasting 4.5 min each. Participants were asked to not play the game more than one time per day (the system prevent them to do it), and if possible, nor wait more than 4 days between sessions. Participants were reminded to complete the game right after completing the session, and were sent another reminder again after 2 days, in the case they did not start the next session. Details of intervention design and session features are summarized on Fig. 3.

Session 1 was supervised remotely, which allowed us to collect relevant information about participants’ interactions with the web platform. Participants had to access the Zoom platform and share the screen with the researcher, who guided them to navigate the platform, including game instructions, 10-min practice, and playing the game. The researcher observed participants’ performance with video and audio off to avoid distractions and was available to clarify any aspects of the task between the trials. In session 1, participants learned to play the game with the tasks in the same screen. In session 2, for which participants had to access the game platform alone and play the game without supervision, the tasks began to be presented in separate screens (split mode). Participants had to press a button to switch between two screens and therefore track the two tasks alternately, imposing a higher cognitive load and increasing the difficulty of the game.

In session 3, the game difficulty increased, since participants had to deal with more food items and apply EmCh manipulations to the game. Participants were instructed to direct their attention to the Cooking task during session 3 as 75% of their scores in each trial would be based on their performance on this task; the emphasis was shifted to the Table Setting task in session 4 with the same instruction. During session 5, emphasis alternated between the Cooking and Table Setting tasks depending on the trial. To ensure clarity of EmCh instructions, session 3 was also supervised remotely through Zoom. Sessions 4 and 5 had the same structure as session 3 but were not supervised; therefore, participants had to apply EmCh when playing the game without supervision.

To reduce attrition and promote retention, the staff could be contacted by email to discuss potential problems, and if necessary, a phone call or extra Zoom session could be scheduled. Reminders were sent through email before every session. Session’s adherence and completion were monitored remotely through the game platform. In case of reduced adherence, such as delay for completing a session within the period suggested, we contacted the participant to identify the reasons and/or barriers and help identify solutions.

#### Game measures

Three outcomes were generated and presented to the participants after each trial: (1) *Number of Correct Tables*, or the number of full tables completed under the correct rule (by tableware or by guest), with higher scores reflecting better performance; (2) *Cooking Time Discrepancy*, the difference between the actual and required cooking time for each food item (averaged across all foods); and (3) *Range of Stop Times*, the difference in stop time between the first and last food item stopped. For outcomes 2 and 3, units represent seconds, and lower scores reflect better performance.

### Implementation outcomes

#### Feasibility

Intervention feasibility was assessed through three outcomes: recruitment, adherence, and retention. (1) *Recruitment:* we examined the recruitment rate, defined as the proportion of approached participants that provided consent and those enrolled in the study. Recruitment characteristics were described in order to understand potential barriers, difficulties and interest of participants to be part of the study. (2) *Adherence and retention:* brief unsupervised remote cognitive training to older adults frequently shows high adherence and retention rates, such as 80% to > 94% [3, 32, 42]. For the present study, we conceptualized intervention adherence and study retention as 80%. Therefore, *adherence* was defined as attendance of at least 4 of the 5 sessions within 4 weeks, and *retention* was conceptualized as the proportion of enrolled participants who completed the study, including post-intervention questionnaire.

#### Acceptability

Acceptability was assessed based on participants’ quantitative and qualitative responses in the *post-intervention questionnaire*. They were asked to provide scores (0 to 10) and their views on specific issues within the BT. For instance, they were asked to provide scores on the difficulty of the game, clarity of instructions, and accessibility of the platform. They were also asked to rate how much they enjoyed the game and their overall experience in the study. Moreover, participants were asked to answer open-ended questions about the game and its influence on their daily life.

#### Game performance and analysis

Game performance was assessed through the scores recorded in each trial in all sessions. Session effect was assessed using a repeated-measures analysis of variance (ANOVA) for each game measure. The effect of EmCh instruction was assessed using one-way ANOVA by grouping trials under the same emphasis (Table Setting or Cooking tasks), regardless of the session. In addition, to compare the variance across games outcomes, we calculated the mean and standard deviation (SD) for each outcome based on the five sessions together and run a repeated-measure ANOVA. Based on these measures we also calculated the Coefficient of Variance (CoV = SD/Mean) for each outcome. Statistical analyses were performed using the Statistical Package for Social Sciences, version 26.

## Results

### Sample characteristics

The mean age of participants was 69.12 years old (SD = 3.83, range 60 to 7 5years), and mean education was 17.66 years (SD = 3.03, range 12 to 25 years). Of the total recruited participants (*n* = 24), 75% were women (*n* = 18), and 95% were white. In addition, all participants were cognitively healthy with a mean MoCA score of 28.66 points (SD = .04, range 27 to 30 points) and lived independently, with a mean ADL-x score of 7.5 points (SD = .88, range 6 to 9 points). The sample sources were (1) the Columbia University *RecruitMe* research platform, and (2) the Bloomingdale Aging in Place (BAiP), a neighborhood not-for-profit organization formed by volunteers and serves older adults living in the Upper West Side area of Manhattan (New York City), and (3) referral from research participants. Participants played the game with laptop or desktop, and all participants used a mouse while playing (smartphones and tablets were not used in the study).

### Feasibility

#### Recruitment

**Fig. 4:**
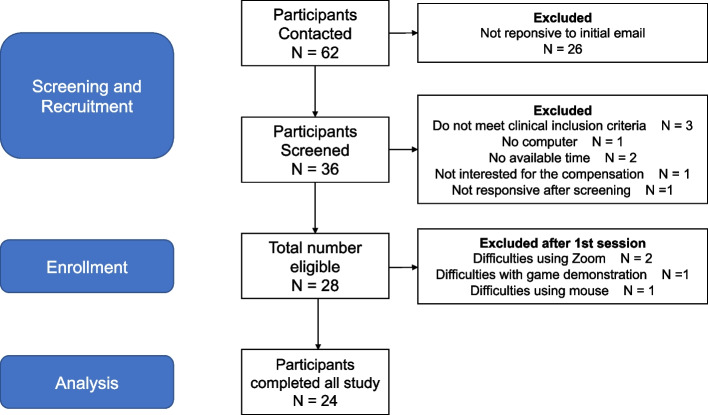
CONSORT flowchart for selection of study participants

Figure 4 outlines the progression of participants from recruitment to post-intervention data collection. The subject flow follows the CONSORT diagram for feasibility and pilot clinical trials [20].

Most participants were recruitment from the online platform RecruitMe and from the organization BAiP. From all 62 participants contacted, 58% (*n* = 36) responded to the initial email. It is worth mentioning that part of these participants was already in our contact list before the COVID-19 pandemic, so it is possible that this influenced the lack of responses. From those that responded to the initial email, 80.5% (*n* = 29) were eligible and consented to participate. Out of these 29 participants, five had to be excluded, resulting in a recruitment rate of 82.7% (24/29). One participant had difficulty with time availability to schedule the first session. The remaining four participants had to be excluded after attending the 1st session due to technical demands, such as difficulties with Zoom platform (*n* = 2), mouse use (*n* = 1), and learning to play the game during practice (*n* = 1). Despite that, all 24 participants who attended the 1st session successfully completed the study. We considered our recruitment process to be feasible and could recruit > 80% of the participants who were eligible and provided consent.

#### Adherence and retention

Our study showed 100% adherence and retention, since all participants that completed the 1st session completed the five sessions and the post-intervention questionnaire. The average amount of days between sessions was 3.3 days (SD = 1.4, range 1 to 9 days), and the average time for completion of the study was 13.3 days (SD = 5.6, rage 7 to 31 days).

### Acceptability

Table 1 shows the responses on the post-intervention questionnaire. Most participants provided high scores when asked if they liked the overall concept of the game and considered the game to be somewhat difficult. Participants report low difficulty in accessing the game online, playing without supervision, and understanding the instructions. Most participants did not consider the game to be childish for their professional level, an aspect which could have interfered in their motivation. Responses were variable when asked if they wanted to continue to play this type of game, but ~ 70% of participants provided scores above 6 when asked how much they enjoyed the sessions and how likely they would be to refer a friend or family member to the study. Participants provided very high scores for friendliness of the research staff, and ~ 80% of participants rated 7 or above for their overall experience in the study. In addition, participants provided their opinions about three open-ended questions: what they enjoyed the most, what was more challenging, and if the game had any impact on their daily life. A summary of these responses is presented in Table 2.

**Table 1:** Post-intervention questionnaire to examine program acceptability

**Acceptability** questionnaire	**Mean (SD)**
1. How much did you like overall concept of the Breakfast Game?	7.79 (1.86)
2. How difficult was the game for you? Which part was particularly difficult?	4.55 (2.16)
3. How difficult was to access the game by yourself (without supervision)?	0.45 (0.88)
4. How difficult was to play the game without remote supervision?	0.37 (0.82)
5. How difficult was to understand the instructions?	0.66 (1.27)
6. How much do you feel the training was too childish for your professional level?	2.62 (3.00)
7. How interested would you be to continue this type of game?	4.95 (3.66)
8. Overall, how much you enjoyed the sessions? Which part you enjoyed the most?	6.91 (2.08)
9. How likely would you be to refer a friend or family member for this study?	7.00 (2.94)
10. How friendly was our staff (How well did we treat you)?	9.79 (0.50)
11. How would you rate your overall experience in the study?	7.91 (1.71)

**Table 2:** Perceptions about the program

**What did you enjoy most?**	**What was challenging?**	**Impact on daily life?**
. Table setting . Setting by either rule . Timing tables with food . Enjoyed seeing how many tables could be made . Testing/competing/challenging against self . Challenge of meeting time	Technical issues . Accidentally highlighting table when trying to drag a utensil . Difficult/clunky to drag & drop utensils precisely . Small space to place utensils, motor difficulties . Instruction text could be bigger Repetitiveness/tediousness . Repetition of thumb movement . Hand/arm soreness . Repetition of same difficulty level Others . Remembering table rule . Meeting cooking time . Feeling anxious to deal several information . Understanding scoring	Mostly no Positive responses: . Improved performance on other online game . Concentrating/focusing more . Figure out best way to do repetitive tasks with least boredom . Thinking more about what is important when dealing with multiple tasks

### Game performance

**Fig. 5:**
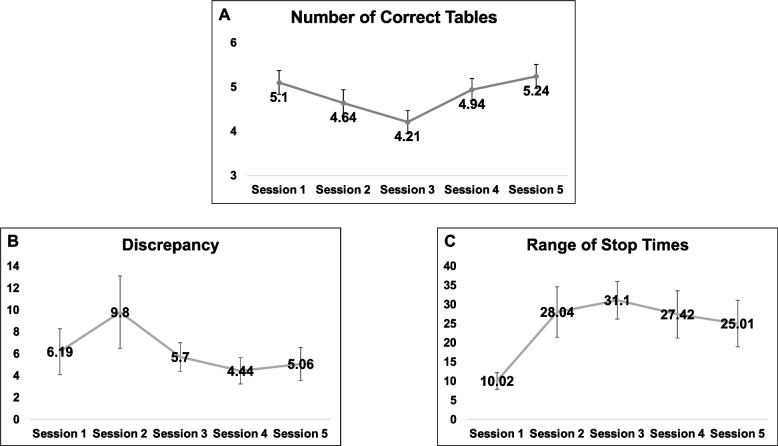
Breakfast Task Training performance across sessions. *Legend*. **A** Higher values represent better performance. **B**,** C** Lower values (sec.) represent better performance. Session 1 and 2: no emphasis change; Session 3: Emphasis on Cooking; Session 4: Emphasis on Table Setting; Session 5: Emphasis on both tasks (random order)

The repeated measures ANOVA revealed a significant session effect for Number of Correct Tables [*F*(4,92) = 10.80, *p* < .001], indicating differences between all five sessions (*p* < .05) (Fig. 5A). The number of tables successfully completed decreased from session 1 to 2, when the split screen mode was introduced, and again decreased from session 2 to 3, when more food items were included in the game. However, in sessions under similar difficulty level (session 3, 4, and 5), the performance linearly improved as a function of session. This trend was even more robust when we excluded sessions 1 and 2 and analyzed sessions 3 to 5 only [*F*(2,46) = 27.53, *p* < .001].

We did not observe a session effect on the Discrepancy [*F*(4,92) = 1.98, *p* = .10], but we observed a session effect on Range of Stop Times [*F*(4,92) = 3.51, *p* = .01] (Fig. 5B, C, respectively). Similar to the pattern observed in the Table Setting task, there was a worsening in the performance from session 1 to 2 and from session 2 to 3 for both Cooking outcomes, and there was an improvement in the performance from session 3 to 5.

It is worth mentioning that the Table Setting performance showed some variability (CoV = 0.22), but was more homogeneous than the performance on the Cooking tasks, which was highly variable, particularly Discrepancy (RST CoV = 1.04, and Discrepancy CoV’s = 1.51).

Regarding EmCh instruction effect, we observed a significant effect of emphasis instruction on Number of Correct Tables [*F*(1,575) = 24.03, *p* < .001]. As expected, better performance on the Table Setting task occurred when emphasis was placed on the Table Setting task. However, there was no emphasis instruction effect on Discrepancy [*F*(1,575) = .94, *p* = .33] or Range of Stop Times [*F*(1,575) = .46, *p* = .49].

## Discussion

The present feasibility study is an initial step in the development of an evidence-based theory-driven cognitive training approach for cognitively healthy older adults. Our pilot data showed that the web-based training platform, the Breakfast Task Training, is feasible in older adults, and the intervention procedures were reported to be acceptable by the participants.

Our results showed overall high recruitment (82.7%), adherence, and retention (100%) rates of the online 5-session protocol. These findings align with those of previous studies conducting brief unsupervised remote cognitive training to older adults, reporting high adherence and retention rates, such as 80% to > 94% [3, 32, 42].

Regarding the game performance, our analysis revealed a learning curve across the sessions, an encouraging observation for a future definitive trial. During the Table Setting task, participants significantly improved their performance, especially across sessions of the same difficulty level. This pattern was also observed on the Cooking task outcomes, albeit only at the trend level since the effect was not significant. The fact the participants considered Cooking task to be harder than the Table Setting task may have influenced this result, and it is likely that additional practice is necessary to show improvement in both tasks, especially within the Cooking task [25]. It is promising that the participants were responsive to EmCh instructions, although its effects were significant for the Table Setting task only. In this feasibility study we applied EmCh in three sessions, which may have not been enough to produce observable effects in both tasks. In a future definitive trial, dose needs to be carefully planned, and additional sessions will allow us to further understand the task learning as well as the EmCh effects in both tasks.

The fact that EmCh manipulation was effective on Table Setting but not on the Cooking is consistent with a previous work with young participants [25]. It is possible that the low variance in Table Setting and the high variance in the Cooking outcomes may have contributed to this finding. The nature of load is very different in Table Setting compared to Cooking, since in the Cooking task there is an embedded conflict between Discrepancy and Range of Stop Times. As no preference instructions were given between the Cooking task rules, participants may have oscillated between the two rules in each round. As a result, we suggest there is already an emphasis decision within each Cooking task trial, which may have difficulted the participants to cope with the emphasis instructions between Cooking and Table Setting tasks. We believe the consequence of this is the very large CoVs observed in the Cooking task outcomes. For future trials, we should carefully review this aspect in order to enhance efficiency of EmCh approach.

It is relevant that participants reported little difficulty in accessing the game online without supervision and in understanding the game instructions. This shows that we were successful in our approach to teach the instructions of the game and how to use the platform at home. In addition, these perceptions are consistent with the high adherence and retention rates of the study. Participants’ responses indicated they liked the concept of the game, and most participants provided medium to high scores when asked if they enjoyed the game and about their overall experience in the study. Some participants reported enjoying competing against themselves and observing their improvement across sessions. Despite that, the responses were variable when asked if they wanted to continue to play this type of game. This is a relevant aspect that should be considered for a future definitive trial. It is possible that some of the technical difficulties and repetitiveness reported may have influenced how much participants enjoy the sessions. Future definitive trial using the Breakfast Task platform should consider structuring the sessions in a more dynamic way, with less repetition across rounds within the same session, advancement of the technical issues reported in this first pilot study.

It is worth mentioning that the BT is cognitively demanding and involves executive control in high load, which is a close simulation of many daily tasks. Therefore, the fact the platform involves an ecological training brings good prospects to its future transfer value, since it simulates similar demands of a common multitasking situation (i.e., preparing a meal/setting tables) and train a strategy that may be applied to different contexts. This was reflected in some of the participant`s qualitative responses after the training, such as feeling they could “think more about what is important when dealing with multiple tasks”, “concentrate/focus more”, “figure out how to do repetitive tasks with least boredom”.

Despite the general good feasibility and acceptability of the BT, difficulties and limitation were noted and should be considered. This study was conceptualized before the COVID-19 pandemic and was adapted to become remote, so the recruitment strategy was originally designed for a remote study. It is worth mentioning that most of our participant source came from a specific organization (*BAiP)* and *RecruitMe* platform during the pandemic, which was not the ideal approach to recruit a diverse sample, since 95% of the sample was highly educated and white. The sample was highly educated and likely presented greater technology proficiency than average, an aspect that may have inflated the results on feasibility and acceptability. Future trials should consider an active recruitment strategy in specific communities to better represent minority groups and individuals with diverse socio-economic status and computer proficiency.

Although most participants recruited did not have difficulties using a computer with internet and the Zoom platform, these technical demands were the main reason for participants to be excluded from the study after the first session. Other source of difficulties stemmed from the technical demands of the game. For instance, during the Table Setting task, participants reported issues when dragging or dropping utensil items (details on Table 2) and considered the space to place the utensils too small, which required higher motor control than usual mouse use. This observation suggests that vision difficulties (even when corrected) and screen size may have influenced the game performance. Although tablets were not allowed for the study, we recognize as a limitation of the study that there was not a screen size requirement for the computers used (e.g., desktops or laptops) and that we did not collect information about computers features, which should be considered and accounted for in a future trial. It is likely that these technical aspects of the game may have contributed to a less unjoyful experience since it added distractions, and difficulties to play the game. In addition, some participants reported the task design was repetitive, which may have caused some tediousness and contributed to the light soreness in the hand/arm. All these game issues should be carefully reviewed and improved for a future trial. Although most of the session’s attendance happened within the study framework, as the average between sessions was 3.3 days, on some occasions participants needed to wait longer periods (i.e., > 4 days) between sessions, with a maximum of 9 days in one case. To the best of our knowledge, the main reasons for the longer wait periods were due to health issues, short trips, unstable internet, or changes in their schedules. Similarly, the average time to complete the study (13.3 days) was within the expected 2 to 3 weeks period; however, there was a large range (i.e., 7 to 31 days). It is possible that the variability observed between sessions and to complete the study could have been mitigated if we had additional reminders or set more strict time limits (between sessions and to complete the intervention). Although we asked participants not to wait more than 4 days between sessions, we did not exclude participants based on attendance in this pilot trial.

The variability in the inter-session interval may happen in any intervention study, and it has the potential to influence intervention response. Different strategies such as additional reminders are relevant in order to engage participants in the study framework. Additionally, it would be beneficial for a future trial to set time limits between sessions or to complete the study. Moreover, strategies for long-term adherence are critical in longer interventions, particularly a dynamic approach that introduces novel aspects of the task in each session, as some participants complained about repetitiveness across rounds and sessions.

In conclusion, the present feasibility study has generated relevant pilot data about the BT training in cognitively healthy older adults and is a promising intervention tool to be incorporated in future trials. It is worth mentioning this is a first step to assess feasibility and acceptability in older adults, and this study was not designed to access efficacy of the proposed training platform. Additional studies are necessary to better understand the efficacy of using the BT training platform and the EmCh approach. In the event that shows to be beneficial, it may be used for clinical purposes in the future. One of the advantages of the BT platform is its ecological approach and flexible interface which allows adaptation of the task difficulty, instructions, dose, duration, and frequency. While the 5-session format was enough to provide initial feasibility, acceptability, and adherence/retention, longer intervention format and control design are necessary to investigate the potential benefits of BT training effects in older adults, including the game outcomes and transfer effects.

## Data Availability

The datasets used and/or analyzed during the current study are available from the corresponding author on reasonable request.
